# Hierarchical clustering analysis of reading aloud data: a new technique for evaluating the performance of computational models

**DOI:** 10.3389/fpsyg.2014.00267

**Published:** 2014-03-31

**Authors:** Serje Robidoux, Stephen C. Pritchard

**Affiliations:** ARC Centre of Excellence in Cognition and its Disorders, Department of Cognitive Science, Macquarie UniversitySydney, NSW, Australia

**Keywords:** computational modeling, reading aloud, hierarchical clustering, non-word reading

## Abstract

DRC (Coltheart et al., 2001) and CDP++ (Perry et al., 2010) are two of the most successful models of reading aloud. These models differ primarily in how their sublexical systems convert letter strings into phonological codes. DRC adopts a set of grapheme-to-phoneme conversion rules (GPCs) while CDP++ uses a simple trained network that has been exposed to a combination of rules and the spellings and pronunciations of known words. Thus far the debate between fixed rules and learned associations has largely emphasized reaction time experiments, error rates in dyslexias, and item-level variance from large-scale databases. Recently, Pritchard et al. (2012) examined the models' non-word reading in a new way. They compared responses produced by the models to those produced by 45 skilled readers. Their item-by-item analysis is informative, but leaves open some questions that can be addressed with a different technique. Using hierarchical clustering techniques, we first examined the subject data to identify if there are classes of subjects that are similar to each other in their overall response profiles. We found that there are indeed two groups of subject that differ in their pronunciations for certain consonant clusters. We also tested the possibility that CDP++ is modeling one set of subjects well, while DRC is modeling a different set of subjects. We found that CDP++ does not fit any human reader's response pattern very well, while DRC fits the human readers as well as or better than any other reader.

Reading aloud involves converting printed character strings into phonological codes. In the case of words, one can rely on memory structures to provide the appropriate pronunciation. However, where novel letter strings are concerned the reader must perform the translation in some other way. One question under considerable debate is whether readers adopt a set of strictly applied rules for this conversion, or if there is a more subtle set of associative relationships between letter patterns and pronunciation at play. The role of grapheme-phoneme conversion rules (or GPCs) and trained, neural networks that learn implicit associations is at the heart of the debate between two of the most broadly successful computational models of reading aloud currently available. When converting printed words into phonology, both the Dual-Route-Cascaded Model (DRC; Coltheart et al., 2001) and the Connectionist Dual-Process models of reading aloud (CDP+/++; Perry et al., 2007, 2010) rely principally on nearly identical lexical systems that store the appropriate information. When it comes to pronounceable non-word letter strings, however, DRC assumes reading is accomplished through the use of GPCs, whilst CDP+ and CDP++ rely instead on a simple neural network that has learned to associate graphemes with phonemes through exposure to a combination of real words and rules.

In debating the relative merits of the two approaches, researchers have relied extensively on experimental results that used reaction times and error rates as the principal variables of interest. On those metrics, CDP++ enjoys an advantage over DRC: it is able to simulate consistency effects, and is able to account for more of the variance in human response times when assessed against large-scale database studies such as the English Lexicon Project (Perry et al., 2007).

While these modal metrics of human behavior are important, they ignore a separate question that is particularly relevant to the debate between strong GPCs such as those in DRC and associative learning algorithms such as the one implemented in CDP++: do the pronunciations produced by the models in response to novel stimuli match those of human readers? In other words, when presented with an item like “PHLOMB,” DRC produces the response 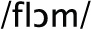,^1^ (as in “bomb”) while CDP++ responds 
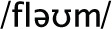
 (as in “comb”). Little research has thus far compared the model responses to those produced by subjects.

Pritchard et al. (2012) examined just this question. They submitted 1475 non-word letter strings made up of onsets and bodies that exist in English, and legal bigrams, to DRC and CDP+/++ and identified 412 that differentiated between the two models. 45 human readers then read these 412 items aloud and their responses were coded for phonology. Comparing the human responses to those of the models, they found that, while both models had some difficulties in matching the empirical data, DRC outperformed CDP+ and CDP++. For these 412 items, DRC was more likely to produce the response most common among the subjects (the modal response), and less likely to produce a unique response that no human reader produced.^2^

## An unanswered question

Pritchard et al.'s (2012) item-by-item analysis clearly favors the view that DRC captures “typical” human non-word reading better than CDP+ or CDP++, but it's difficult to know what “typical” means here. Subjects vary considerably in the kinds of responses they produce. Whereas some non-words produced 100% agreement among the subjects, other non-words resulted in up to 24 different responses. This difficulty led Rastle and Coltheart (1999) to define the DRC's goal as producing the modal response for all items:

“All we seek to achieve is that for all non-words, the DRC model's pronunciation is the one that the majority of readers assign.” (p. 484)

However, it is evident even from the Pritchard et al. (2012) data that this is not always possible: twelve items produced more than one possible modal response (e.g., SLYS was pronounced as “sleece,” “slice,” and “sleeze” by 12 readers each). In other cases, though there was one true mode in the sample, there was often a very near-modal alternative response (e.g., CESH is pronounced as “sesh” by 19 subjects, and “kesh” by 20 subjects). For such items, choosing the target response according Rastle and Coltheart's (1999) goal is not as unambiguous as it might at first seem.

An alternative (and probably complementary) approach to evaluating the model success is to compare subject response *profiles* against each other and against the models to determine whether there are different groups of subjects with similar response profiles, and whether the models perform better at fitting some of these groups over others. Looking at overall profiles rather than item-by-item analyses allows us to ask two questions: first, are there clusters of subjects that tend to respond similarly in a way that is not readily detected by the item-by-item approach. Second, are there some subjects who seem to match the DRC's GPC-driven responses while others tend to use the more fluid associations learned by CDP+/++? Answering this question requires a way to simultaneously compare all subjects and the models on their overall response profiles, and not on an item-by-item basis. Here we discuss one approach to this problem.

### Hierarchical clustering

Hierarchical clustering techniques are designed to do just this. Conceptually, hierarchical clustering^3^ is a simple algorithm:

1. For each possible pair of subjects, produce an index of how (dis)similar they are. This is the distance matrix.
2. Starting with each subject as an individual cluster.
3. Merge the two nearest clusters, recording the distance between them.
4. Repeat step 3 until all subjects have been merged into a single cluster.

**Figure 1:**
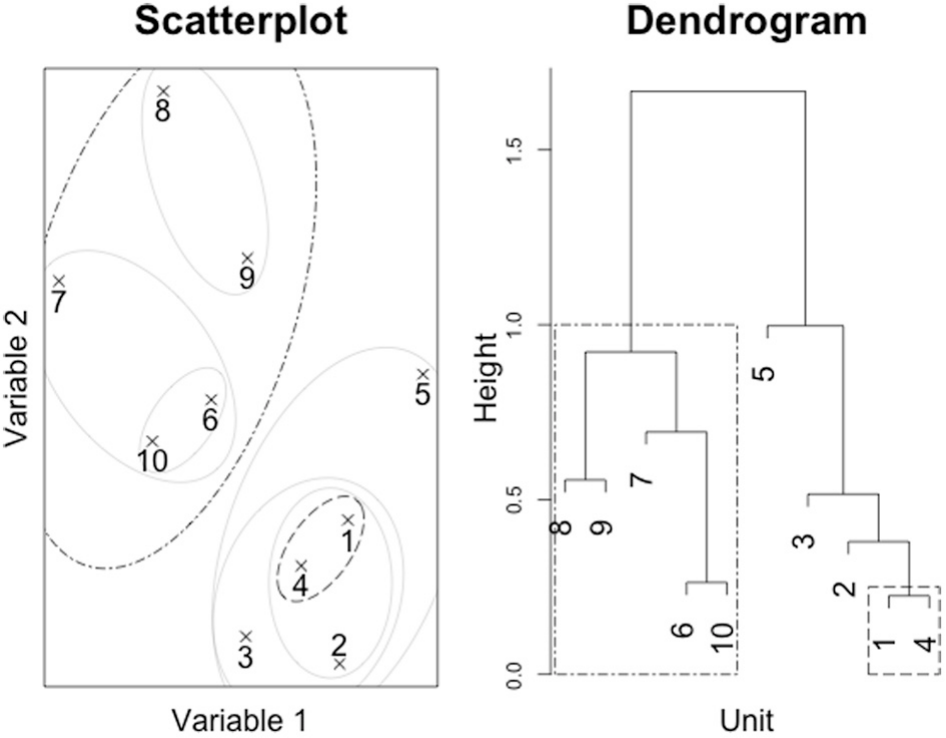
**Clustering of artificial data and dendrogram**. Light gray ellipses indicate clusters, while the dark, dotted ellipses indicate the corresponding clusters in the dendrogram.

This converts a set of data into a series of cluster mergers along with the distances between the merged clusters (see Figure 1 for an illustration using two-dimensional, real-valued data).

The relationship among the distances and clusters can be depicted in a dendrogram. Figure 1 illustrates the process using artificial two-dimensional data (depicted on the left). The resulting dendrogram is depicted on the right. Each horizontal line merges two subclusters, while the height at which the horizontal line is drawn reflects the distance between the two clusters being merged. In this simple dataset, it is easy to see that subjects 1 through 5 and subjects 6 through 10 form two distinct clusters. The subjects within the clusters tend to be joined at small distances (merged at lower points in the figure), while the two distinctive clusters are further from each other (indicated by the high merge in the graph). One can also see that subjects 1 and 4 are nearest each other in the scatterplot and are merged at the lowest point in the dendrogram (at a height of approximately 0.2; enclosed in the smaller dotted box to the right). The clusters represented by subjects {6, 7, 10} and {8, 9} are further from each other, and are thus merged higher on the dendrogram (at approximately 0.9; see the larger dotted box on the left).

#### Clustering methods

**Figure 2:**
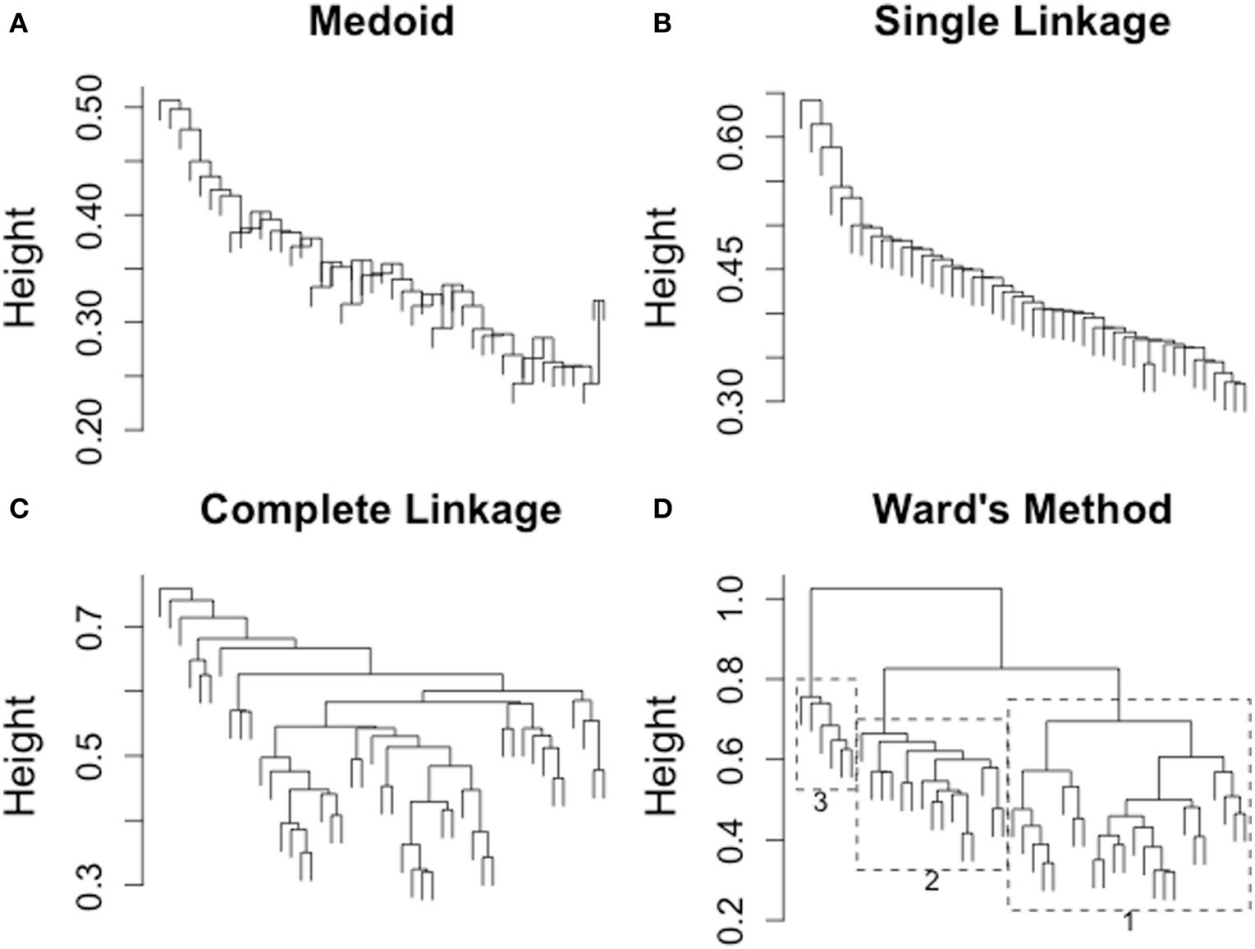
**Results of applying different clustering methods to subject data from Pritchard et al. (2012)**. These dendrograms do not include model responses. Methods depicted are **(A)** medoid, **(B)** single linkage, **(C)** complete linkage and **(D)** Ward's method.

The clustering algorithm requires a definition of “distance” between not only individual subjects, but also clusters of subjects. In the case of individual subjects, this distance is determined by step 1 above. For numerical data, some form of scaled Euclidean distance is often used (categorical data will be discussed further on). However, there are many options for defining the distance between groups of subjects. The most commonly used approaches are Medoid/Centroid, Single Linkage, Complete Linkage, and Ward's method. These four methods are briefly described here, but a full treatment of clustering methods is beyond the scope of this article. The interested reader can find these techniques described in detail in cluster analysis texts such as Everitt et al. (2011). Figure 2 depicts the results of applying them to the subject responses in Pritchard et al.'s (2012) study.

#### Medoid/centroid

For each cluster, the medoid or centroid is a typical element of the group. A centroid is a theoretical element that has the mean cluster value for each variable that contributes to the dissimilarity calculation. This element is likely not an actually observed element. The medoid is the individual element that is, on average, closest to all of the other elements in the cluster (in a sense the existing element that best “represents” the whole cluster).

#### Single linkage

The distance between two clusters A and B is defined as the smallest distance between any element in cluster A and any element in cluster B. This is sometimes referred to as the “friends-of-friends” approach, since it can result in long chains of single elements being merged into the larger cluster (Figure 2B).

#### Complete linkage

This is the complement of the single linkage approach. The distance between two clusters A and B is defined as the *largest* distance between any element in cluster A and any element in cluster B. This approach ensures that the distance between every pair of elements in the two clusters are contained within the distance between the two clusters (see Figure 2C).

#### Ward's method

Unlike the other methods described above, Ward's method does not rely on a distance metric analogous to the one used to determine the matrix in step 1 above. Instead, Ward's method minimizes the mean squared distances within the groups. At each merger, Ward's method identifies the two clusters whose merger would have the smallest influence on the mean squared within-cluster distances. Ward's method is biased toward producing spherical clusters (in essence clusters of roughly equal size; see Figure 2D).

The principal goal of clustering techniques is to uncover structure that may be hidden in complex data. Since this is inherently exploratory, the method that produces the most distinctive clusters in a particular data set is typically the one selected. Once clusters have been identified, a closer look at the variables that distinguish clusters from each other is necessary to determine the nature of the structure.

## Clustering reading aloud data

When the data being used for clustering is numerical, there are any number of approaches to defining the distance between elements. Euclidean distances between elements (using normalized variables to avoid scale effects) are common. However, in the Pritchard et al. (2012) study, the data are reading aloud responses to 412 non-word items. Such datasets are categorical in nature. In the case of categorical data, a pair of subjects either match or do not match on each variable. Here we opt to define the distance between two subjects as the percentage of items on which the two subjects' responses disagreed. According to this metric, a distance of 0.3 between two subjects would indicate that the subjects disagreed on 30% of the items in the Pritchard et al. dataset.

Hierarchical clustering offers us a way to simultaneously compare Pritchard et al.'s (2012) subjects across all 412 items to uncover groups of subjects that tend to be similar in their response profiles. If such latent structure can be uncovered, a closer look at the responses can help us to understand how subjects differ from each other. Further, by treating responses from computational models as theoretical subjects, we can compare the DRC and CDP++ models to the human subjects and see whether some subjects tend to cluster with one model or the other.

### Human readers

Figure 2 depicts the results of clustering the Pritchard et al. (2012) data (subjects only) using each of the four clustering methods previously described. The first three methods (Figures 2A–C) provide little in the way of clusters for further evaluation. Ward's method (in Figure 2D) offers some evidence that there may be structure hidden among the subjects. Three distinct groups emerge. In Figure 2D, the clusters are delineated by light gray boxes and labeled in order of the size of the cluster (so that cluster 1 is the largest, and cluster 3 the smallest). Cluster 3 consists of a small subgroup of anomalous readers who are not particularly similar to each other or anyone else, while clusters 1 and 2 seem to offer more internal consistency.

#### Distinguishing the clusters

The power of clustering is in its ability to uncover structure that isn't based on a priori groupings (such as readers who produce regular pronunciations for non-words vs. those who produce irregular pronunciations). However, such structure is only useful if the two groups can be differentiated on the basis of their responses in some way. To determine whether and how these two groups differed, we examined the individual items by cluster and identified one possibility: the two primary groups do differ in their affinity for regularizations, but only for select ambiguous graphemes. Specifically, it seems to be a small set of ambiguous *consonant* graphemes that drive most of the difference between subject clusters.

Table 1 summarizes the types of items that underlie at least part of the difference between the two largest clusters of subjects. Consonant graphemes containing “C” are particularly discriminating, with non-words beginning with CE, CI, or CH, or ending with CE, CH, or CHE all producing different response patterns in the two clusters. “C” is not alone in discriminating clusters, however, as non-words beginning with “PH,” beginning or ending with “GN,” and non-words using “Y” as the only vowel cluster also discriminated. Some general observations follow.

**Table 1:** **Pronunciations that distinguished between subject clusters 1 and 2**.

**Pronunciation of C in CE- Items**					
**Cluster**	***s***	***k***	***t*∫**		
1	79.7	14.0	6.3		
2	46.1	52.8	1.1		
3	44.4	55.6	0.0		
**ITEMS: CERM CEBB CELK CES CEB CESH**					
**Pronunciation of C in -CE Items**					
**Cluster**	***s***	***k***	***t*∫**	**∫**	**Other**
1	83.9	3.4	9.9	2.5	0.3
2	75.4	10.1	5.5	7.0	2.0
3	69.9	6.0	7.2	8.4	8.4
**ITEMS: LARCE HACE PHLAUCE WAICE BLAUCE SKARCE WAUCE PHLEUCE CICE**					
**Pronunciation of C in CI- Items**					
**Cluster**	***s***	***k***	***t*∫**	**Other**	
1	91.4	5.7	2.9	0.0	
2	61.4	34.1	2.3	2.3	
3	50.0	44.4	0.0	5.6	
**ITEMS: CICE CILTH CID**					
**Pronunciation of PH in PH- Items**					
**Cluster**	***f***	***p***			
1	99.0	1.0			
2	88.8	11.2			
3	97.2	2.8			
**ITEMS: PHLAUCE PHOMP PHLOMB PHRALPH PHOL PHONK PHOLK PHLEUCE PHOIN PHLOSE PHUGE PHLOTH PHUISE PHROOK PHLERSE PHLOLT PHEASE PHOZ**					
**Pronunciation of CH in CH- Items**					
**Cluster**	***t*∫**	***k***	∫	***s***	**Other**
1	80.9	4.3	12.2	1.7	0.9
2	67.1	15.1	5.5	8.2	4.1
3	64.3	25.0	3.6	0.0	7.1
**ITEMS: CHONGE CHIEL CHYNCH CHUILT CHACH**					
**Pronunciation of CH in -CH Items**					
**Cluster**	***t*∫**	***k***	***s***	**Other**	
1	80.8	6.4	10.5	2.3	
2	72.4	14.1	10.6	2.9	
3	64.3	18.6	13.2	3.9	
**ITEMS: ELCH THWONCH SMYNCH GRACH JEICH PSAUNCH GHELCH GEECH CHYNCH FRECH GYNCH THETCH STAITCH KNOUCH PSICH CHACH BLYNCH NACH GRELCH THANCH WEICH SPLACH**					
**Pronunciation of CH in -CHE items**					
**Cluster**	∫	***t*∫**	***k***	**Other**	
1	66.1	27.3	6.1	0.5	
2	35.8	59.1	4.3	0.8	
3	41.1	43.0	8.4	7.5	
**ITEMS: ROUCHE BOUCHE PLAUCHE DECHE THECHE DAUCHE SNICHE SKECHE SHECHE WHAUCHE VACHE BLAUCHE WRICHE FROCHE SPLICHE DRICHE SMOCHE CRICHE**					
**Pronunciation of Y in -Y- Items**					
**Cluster**	**I**	**i**	**aI**	**Other**	
1	57.2	22.2	19.8	0.8	
2	55.3	30.4	13.0	1.2	
3	27.7	63.1	6.2	3.1	
**ITEMS: SMYNCH SCRYM NYTH CHYNCH SLYS GYNCH SMYS SMYNC FRYMPH BLYNCH GNYTH**					
**Pronunciation of GN in GN- Items**					
**Cluster**	***n***	**[gn]/** 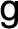 ǝ 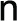 ]	***g***	***kn***	
1	79.3	18.1	1.6	1.1	
2	54.7	35.9	8.5	0.9	
3	27.1	50.0	18.8	4.2	
**ITEMS: GNANC GNEUTH GNOOSH GNUSE GNOMB GNALPH GNOSE GNYTH**					
**Pronunciation of GN in -GN Items**					
**Cluster**	***n***	**[gn]** 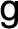 /ǝ 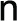 ]	**[ŋ]/[ndƷ;]/[ŋg]**	**Other**	
1	84.5	5.6	8.5	1.4	
2	56.8	9.1	31.8	2.3	
3	33.3	16.7	33.3	16.7	
**ITEMS: VIGN BLIGN GHIGN**					

***Non-words beginning with CE or CI***. For these items, subjects in cluster 1 strongly preferred to pronounce “C” with the regular /s/ over other pronunciations (85% of trials), while subjects in cluster 2 split their responses between /s/ (53%) and /k/ (43%).

***Non-words ending with CE***. Here again, cluster 1 subjects showed a slightly stronger preference for the regular /s/ than did subjects in cluster 2 (84% vs. 75%). What is noteworthy for these non-words is what the subjects in each cluster chose as an alternative to the /s/: cluster 1 subjects chose to infuse the item with some Italian flavor and used /**t∫**/ (10%) while subjects in cluster 2 again preferred the /k/ alternative (10%). Subjects also often read these items as disyllabic (e.g., reading CICE as 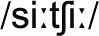). This change in syllabic parsing did not discriminate the clusters.

***Non-words beginning with CH***. Cluster 1 subjects again preferred the regular /t**∫**/ pronunciation here (81%), or alternately a softer /**∫**/ (12%). Cluster 2 subjects also chose the regular pronunciation 67% of the time, but they were much more likely to choose an alternate, either /k/ (15%) or, less commonly, /s/ (8%). Though there is a difference in the tendency to regularize, the distinction between clusters here seems to be in the alternative pronunciations chosen.

***Non-words ending with CH***. Here both clusters tended to strongly prefer the regular /t**∫**/ pronunciation (81% for cluster 1 and 72% for cluster 2). Again, the difference between clusters is highlighted by the alternative pronunciations with cluster 2 subjects more likely than cluster 1 subjects to choose /k/ (14% vs. 6%).

***Non-words ending in CHE***. For these items, cluster 1 subjects preferred the regular /**∫**/ 66% of the time, opting for /t**∫**/ 27% of the time. Cluster 2 subjects showed the opposite pattern, opting for the regular pronunciation only 36% of the time, and preferring the irregular /t**∫**/ 59% of the time.

For some items, cluster 2 subjects seemed to have a preference for simplifying complex or unusual graphemes. Three examples that discriminated the clusters follow.

***Non-words beginning with GN***. Cluster 1 subjects strongly preferred the regular /n/ for this grapheme (79% of trials), only splitting the letters into two graphemes 18% of the time (producing either /gn/ or 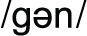). Cluster 2 subjects split the graphemes much more frequently (36% of trials).

***Non-words beginning with PH***. Cluster 1 subjects nearly uniformly chose the regular /f/ for this grapheme (99% of trials), while cluster 2 subjects occasionally seemed to ignore the H or treat it as silent, and produced /p/ on 11% of trials.

***Non-words ending with GN***. Here, cluster 2 subjects frequently produced responses more consistent with reversing the final phoneme. That is, they chose to pronounce the final phoneme as /ŋ/,/ndƷ/, or /ŋg/ 32% of the time rather than as the regular /n/.

Finally, Y was the only vowel that distinguished between the clusters, though not in a simple “regular vs. irregular” way.

***Non-words with Y as the vowel***. Both clusters were equally likely to choose the regular 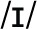 (approximately 56% of trials). However, if subjects chose an alternate response, cluster 2 subjects were slightly more likely to choose /**i**/ (30% vs. 22%) while cluster 1 subjects were more likely to choose 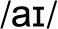 (20 vs. 13%).

### Discussion

It is tempting to characterize cluster 1 and 2 subjects as “regularizers” and “non-regularizers,” respectively. To some extent, this may be a fair classification, but it is tempered somewhat by observations with other graphemes. First, it is noteworthy that the differences between clusters 1 and 2 do not involve *vowel* pronunciations. This is surprising as most discussion of irregularity tends to be weighted toward vowel clusters since these are generally less consistent in their pronunciations (e.g., Andrews and Scarratt, 1998; Jared, 2002). The Pritchard et al. data are consistent with the view that vowels are important to differences in responses, in that many alternate responses differed in the vowels. What the present analysis suggests is that subjects aren't naturally grouped by their vowel pronunciations. Even in the one exception to this observation (when Y is the vowel), they are not distinguished along regular/irregular lines, but rather by their choice of irregularization. To the best of our knowledge, no one has specifically examined irregularity in consonant pronunciations.

It is also not the case that the clusters can be characterized as “consonant-regular” vs. “consonant-irregular.” Many ambiguous consonant graphemes do not distinguish between the two clusters at all. Table 2 summarizes several other consonant graphemes where both cluster 1 and cluster 2 subjects showed similar patterns of regularization. That is, cluster 1 subjects are only regularizers with respect to some graphemes and not others. For example, when considering the grapheme PS at the beginning of words, they are just as likely as cluster 2 subjects to choose similar irregular pronunciations.

**Table 2:** **Pronunciation of other ambiguous consonant clusters that might be thought to distinguish clusters 1 and 2, but do not**.

**Pronunciation of SC in SC- Items**					
**Cluster**	***sk***	***s***	**∫**		
1	91.4	6.5	2.2		
2	92.4	6.4	1.2		
3	90.3	9.7	0.0		
**ITEMS: SCRUKE SCRYM SCAQUE SCROLK SCRIPE SCRALL SCROSE SCUTE SCINE SCROME SCILTH SCRALK**					
**Pronunciation of PH in -PH Items**					
**Cluster**	***f***	***pf***	***p***	***v***	
1	74.5	21.3	4.3	0.0	
2	74.6	16.9	6.8	1.7	
3	75.0	20.8	4.2	0.0	
**ITEMS: FRYMPH TWALPH GNALPH ZALPH PHRALPH**					
**Pronunciation of PS in PS- Items**					
**Cluster**	***s***	**[ps]/[pǝs]**	***sp***	**Other**	
1	73.7	23.9	0.5	1.9	
2	70.2	21.4	3.2	5.2	
3	16.5	40.8	4.9	37.9	
**ITEMS: PSOOSH PSAUNCH PSAWP PSORB PSIRP PSEUCE PSAUGE PSICH PSIZ PSAR PSAISE PSAMB PSONGE PSOATH PSOOTH PSEEF PSEN PSELSE**					
**Pronunciation of NG in -NGE Items**					
**Cluster**	**ndƷ**	ŋ	**ŋdƷ**	**ŋg**	**Other**
1	89.4	5.6	0.7	2.0	2.2
2	91.4	2.5	0.0	4.0	2.1
3	76.9	6.7	1.5	6.0	9.0
**ITEMS: STRONGE CHONGE DONGE THWINGE SHRUNGE ENGE NENGE RENGE SNENGE SNONGE PLENGE KUNGE RINGE FRONGE YOUNGE RHINGE ZENGE PSONGE PLANGE SWOUNGE WROUNGE DANGE THINGE**					
**Pronunciation of TH in TH- Items**					
**Cluster**	θ	***t***	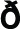	**Other**	
1	97.6	1.5	0.5	0.4	
2	96.8	3.1	0.0	0.2	
3	93.7	4.2	0.4	1.7	
**ITEMS: THAC THEEL THAQUE THWONCH THEDGE THECHE THOLVE THUBE THWUILT THEIL THITE THWAZZ THUSE THRANC THODD THALC THWALC THWINGE THET THESS THAG THELM THETCH THROUSE THELK THAK THWOLVE THWELVE THWOWN THRALC THEL THRUME THREAR THWOS THANCH THESK THERP THWEB THINGE THUPE**					
**Pronunciation of TH in -TH Items**					
**Cluster**	**θ**	***t***	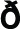	**Other**	
1	97.6	0.0	2.2	0.3	
2	97.0	0.4	1.7	0.9	
3	91.6	0.0	1.1	7.4	
**ITEMS: SHOWTH NYTH GNEUTH STRATH FATH COWTH CILTH LOOTH SPEWTH SMOUTH PHLOTH WREWTH SCILTH PSOATH PSOOTH GNYTH**					

We turn now to a different application of the clustering algorithm. In this second analysis, we ask whether DRC and CDP++ models are better at fitting some subjects over others. Since DRC is, unsurprisingly, highly regular in its pronunciations it comes as no surprise that we would expect it to fit better with subjects from cluster 1 than from the other clusters. CDP++, on the other hand, may be better able to model subjects that tend to choose alternative pronunciations.

### Computational models and human readers

Pritchard et al. (2012) compared DRC, several versions of CDP+, and CDP++ to the human response sets. The various versions of CDP+/++ tended to have very high agreement with each other. Since including several versions of CDP+/++ would induce an artificial cluster, the most successful version of the model (CDP++) could find its results dragged down by the poorer performance of the other models that it resembles. Since CDP++ had the most success in Pritchard et al.'s (2012) analysis, we include it without its siblings in our clustering analysis. This should give CDP++ the best chance of success.

**Figure 3:**
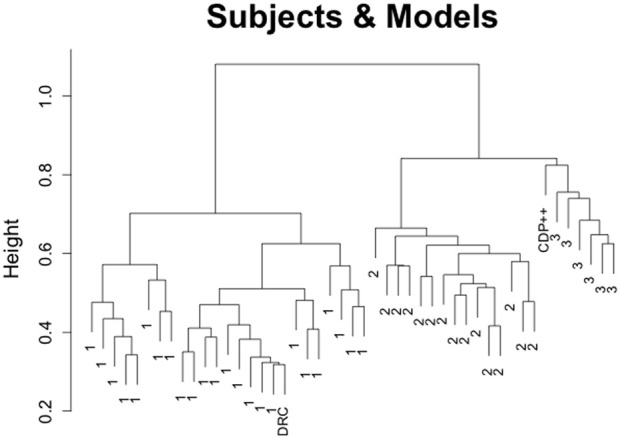
**Clustering results for the Pritchard et al. (2012) non-word reading data, using Ward's method and including response data from the 45 subjects, DRC, and CDP++**.

The results from this clustering analysis are depicted in Figure 3. Subjects are labeled according to their cluster assignment from the previous analysis (excluding the models). This analysis produces two important conclusions. First, it confirms Pritchard et al.'s (2012) finding that DRC matches the responses of subjects more closely than CDP++. In the case of the clustering analysis, DRC is merged into the largest, and most homogeneous cluster of subjects (cluster 1). This suggests that DRC does an effective job of capturing the responses of a large number of subjects, allowing for some variability within and between subjects. Unsurprisingly, these are the subjects that tended toward regular pronunciations of those graphemes that distinguished the cluster 1 from cluster 2 above. It is also worth noting that DRC is merged at the lowest point in the graph. This means that no two subjects are more similar to each other than DRC is to at least one subject^4^. Second, not only is CDP++ underperforming DRC, it performs quite poorly in general, failing to match response profiles with *any* subjects and being relegated to a small group of “hermit” readers who also do not match well with any other subjects (indicated by the relatively high merge distances between and among them)^5^.

### Discussion

DRC and CDP++ are both dual-route models, and thus share many similarities. They also both perform generally well across a range of empirical benchmarks. Adjudicating between the two models now involves closer scrutiny of individual benchmarks, and it appears that each model has a relative advantage over the other. When adjudicating between CDP++ and DRC, it would seem that different analyses arrive at different conclusions. When considering mean reaction time and accuracy data, CDP+/++ enjoys a distinct advantage over DRC because of its ability to simulate consistency effects. CDP+/++ also captures more item-level variance for words (Perry et al., 2007, 2010). However, when comparing responses directly to those produced by subjects, DRC has the upper hand. It's not clear what is at the root of this dissociation. It could be that DRC needs a more flexible set of rules and more fluidity in the possible responses in order to capture more effects and more of the item-level variance. Similarly, it may be that adjustments to CDP++'s training algorithm would allow it to learn a set of associations that more closely reflects those that subjects adopt. As things stand now, neither is clearly dominant across all of the important benchmarks for the computational modeling of reading aloud behavior.

## Conclusion

Hierarchical clustering offers researchers a way to compare subject profiles across a range of variables. In the present study, we illustrate how hierarchical clustering of the reading aloud data from Pritchard et al. (2012) can answer two questions: first, we identified two groups of subjects who differed in their pronunciation patterns. Further, *post-hoc* examination of these clusters identified a few select consonant graphemes that underlie the difference. Critically, the differences did not conform cleanly to “regular vs. irregular” divisions. Second, we were able to provide converging evidence that DRC tends to match subjects better than CDP++. Importantly, we extend those conclusions in two ways: first DRC cannot improve much as a model of a typical skilled reader, since it fits other subjects at least as well as other subjects fit one other. In other words, the heterogeneity among subjects can never be captured by a model of an average reader that does not simulate individual differences between readers. Second,CDP++ does not appear to match any of Pritchard et al.'s 45 subjects very well, challenging a critical component of the model. The inclusion of learning algorithms to broaden a model's scope from simulating skilled reading to simulating reading acquisition may well be an important step forward (Perry et al., 2007), but CDP++ does not appear to be learning what human readers learn. Though no explicit learning algorithms are included in DRC, it appears that the rule system embedded in the GPC sublexical system better captures what skilled readers have learned about the relationship between letters and sounds.

### Conflict of interest statement

The authors declare that the research was conducted in the absence of any commercial or financial relationships that could be construed as a potential conflict of interest.

## Footnotes

^1^The notation used here is based on the International Phonetic Alphabet.

^2^Both DRC and CDP++ are in agreement on pronunciation for most nonwords. Consequently, it would be difficult to draw many conclusions from a broad set of items. These 412 items amplify the differences between the models in order to better adjudicate between them.

^3^The approach described here is more accurately called agglomerative hierarchical clustering, as it starts with many clusters and ends with one. Divisive and non-hierarchical techniques are not considered.

^4^In a separate analysis we included a perfectly “modal” model. That is a hypothetical subject that always gave the modal response to each item. This model was clustered with cluster 1 and DRC, and tended to fit a few subjects better than DRC fit any subjects. That is, some subjects do appear to be more “typical” than DRC is, but DRC still performed quite well.

^5^Note that when we say that CDP++ performs poorly relative to DRC, we mean on these items. These items were specifically selected to amplify the differences between the two models so that we could more closely examine the assumptions that underlie the two models.
